# Enhancing discharge decision-making through continuous monitoring in an acute admission ward: a randomized controlled trial

**DOI:** 10.1007/s11739-024-03582-y

**Published:** 2024-04-15

**Authors:** Niels Kant, Sjoerd H. Garssen, Carlijn A. Vernooij, Gert-Jan Mauritz, Mark V. Koning, Frank H. Bosch, Carine J. M. Doggen

**Affiliations:** 1 Clinical Research Center, Rijnstate Hospital, Wagnerlaan 55, 6815 AD Arnhem, The Netherlands; 2 Department of Health Technology and Services Research, Faculty of Behavioral, Management and Social Sciences, Technical Medical Centre, University of Twente, Hallenweg 5, 7522 NH Enschede, The Netherlands; 3 Department of Anesthesiology, Rijnstate Hospital, Wagnerlaan 55, 6815 AD Arnhem, The Netherlands; 4 Department of Patient Care and Monitoring, Philips Research, High Tech Campus 34, 5656 AE Eindhoven, The Netherlands; 5 Department of Emergency Medicine, Rijnstate Hospital, Wagnerlaan 55, 6815 AD Arnhem, The Netherlands; 6 Department of Internal Medicine, Rijnstate Hospital, Wagnerlaan 55, 6815 AD Arnhem, The Netherlands; 7 Department of Internal Medicine, Radboudumc, Geert Grooteplein Zuid 10, 6525 GA Nijmegen, The Netherlands; 8 Department of Health Technology and Services Research, Technical Medical Centre, University of Twente, P.O. Box 217, 7500 AE Enschede, The Netherlands

**Keywords:** Wearable electronic device, Length of stay, Patient discharge, Randomized clinical trial, Monitoring/physiologic, Clinical decision rules

## Abstract

In Acute Admission Wards, vital signs are commonly measured only intermittently. This may result in failure to detect early signs of patient deterioration and impede timely identification of patient stability, ultimately leading to prolonged stays and avoidable hospital admissions. Therefore, continuous vital sign monitoring may improve hospital efficacy. The objective of this randomized controlled trial was to evaluate the effect of continuous monitoring on the proportion of patients safely discharged home directly from an Acute Admission Ward. Patients were randomized to either the control group, which received usual care, or the sensor group, which additionally received continuous monitoring using a wearable sensor. The continuous measurements could be considered in discharge decision-making by physicians during the daily bedside rounds. Safe discharge was defined as no unplanned readmissions, emergency department revisits or deaths, within 30 days after discharge. Additionally, length of stay, the number of Intensive Care Unit admissions and Rapid Response Team calls were assessed. In total, 400 patients were randomized, of which 394 completed follow-up, with 196 assigned to the sensor group and 198 to the control group. The proportion of patients safely discharged home was 33.2% in the sensor group and 30.8% in the control group (*p* = 0.62). No significant differences were observed in secondary outcomes. The trial was terminated prematurely due to futility. In conclusion, continuous monitoring did not have an effect on the proportion of patients safely discharged from an Acute Admission Ward. Implementation challenges of continuous monitoring may have contributed to the lack of effect observed. *Trial registration*: https://clinicaltrials.gov/ct2/show/NCT05181111. Registered: January 6, 2022.

## Introduction

In most Dutch hospitals, patients are transferred to an Acute Admission Ward (AAW) [1], also known as an Acute Medical Unit, for specific treatment or further observation after presentation at the Emergency Department (ED). In the AAW, intermittent vital sign monitoring and Early Warning Scores (EWSs) are used to assess patient stability and the risk of deterioration [2, 3]. Patients who have achieved a state of stability and do not require additional hospital-based care are eligible for discharge to their homes. Conversely, patients whose condition deteriorates or necessitates extended medical attention are admitted to an in-hospital ward. Physicians aim to determine those eligible for discharge by analyzing vital sign measurements, among other relevant parameters, within the initial 48 h following admission to the AAW. However, while for several patients the next destination after the AAW is clear, this remains uncertain for others, potentially leading to unnecessary and prolonged hospital admissions.

Assessing patient stability and the risk of deterioration at an early stage might aid physicians in making timely and more certain discharge decisions, potentially leading to a reduction in hospital Length Of Stay (LOS) and unnecessary hospital admissions. Currently, vital signs such as heart rate (HR) and respiratory rate (RR), are only measured intermittently, which potentially causes early signs of patient deterioration to be missed [4, 5]. However, continuous vital signs monitoring, which can be facilitated by wearable sensors [6, 7], has the potential to enable earlier determination of patient stability and to detect deterioration during unobserved intervals within intermittent monitoring and thereby support the discharge decision-making process [4, 8, 9].

Therefore, the objective of this trial was to evaluate the impact of adding continuous monitoring of HR and RR by a wearable sensor to usual care on the proportion of patients that can be discharged home safely from the AAW. Only patients who, at the point of being admitted to the AAW, had an uncertain destination after AAW stay were included. Unplanned readmissions, revisits to the ED, and mortality rates within 30 days were assessed to determine whether a patient was safely discharged. Additionally, LOS at the AAW, LOS at other in-hospital wards and total hospital LOS, the number of Intensive Care Unit (ICU) admissions and Rapid Response Team (RRT) calls were assessed.

## Methods

### Trial design and setting

This monocenter randomized controlled trial (NCT0518111—ClinicalTrials.gov) was conducted in the AAW of Rijnstate Hospital, Arnhem, The Netherlands. A detailed protocol has been published elsewhere [10]. Hence, in the current article, the methods are described briefly. Participants were 1:1 randomized into a sensor or control group, based on a pre-determined randomization scheme. This allocation was concealed for both the patient and the nurse, after which the trial was open-label.

### Participants and recruitment

All patients planned for AAW admission after ED presentation were screened for inclusion and exclusion criteria. Typically, patients from all specialties could be admitted to the AAW for a maximum of 48 h, unless an exclusion criterium was met (Table 1). The inclusion criteria were: (1) age ≥ 18 years, (2) ability to read and speak the Dutch language, and (3) an uncertain destination after AAW admission, which was determined by a negative response from the ED physician to both of the following two questions: Is it certain that the patient will be discharged home today or tomorrow? Is it certain that the patient needs an admission to the hospital for longer than 48 h, which would require a transfer to an in-hospital ward following their stay in the AAW? Exclusion criteria were: (1) scheduled surgery within the next 30 days, because this affects both the LOS and readmission rates, regardless of the patient's clinical condition throughout the AAW stay, (2) known pregnancy or breastfeeding, (3) possession of an active implantable device, (4) presence of any skin condition that hinders placement of the wearable sensor, (5) known sensitivity to medical adhesives, (6) use of creams or lotions that affect the skin in the area where the wearable sensor is positioned. To address distinct research objectives beyond the scope of the present article, wearable sensors remained attached for 14 days, leading to the following additional exclusion criteria: (7) intent to visit a sauna or engage in swimming activities within the next 14 days, (8) inability or unwillingness to wear a wearable sensor for a period of 14 days. Upon meeting all the eligibility criteria, informed consent was obtained from a participant by a nurse in the AAW.

**Table 1:** Exclusion criteria for admission to the Acute Admission Ward of Rijnstate hospital

General	Patients who need to be admitted to the Cardiac Care, Medium Care or Intensive Care Unit Patients requiring specific psychiatric care Patients under the age of 18 Patients with multiple fractures Patients who require isolation measures for tuberculosis, Methicillin-resistant Staphylococcus Aureus and Varicella Zoster Post examination recovery patients
Internal medicine	Patients with lymphatic leukaemia Patients with Human Immunodeficiency Virus infection who require treatment Patients who require dialysis Patients with a renal transplant Patients with autologous stem cell transplantation within the last 6 months
Pulmonology	Patients who require non-invasive ventilation Patients who require > 5 L/minute of supplemental oxygen Patients who are unstable after 2 h of oxygen supply via a non-rebreathing mask
Gastroenterology	Patients with pancreatitis Patients with gastrointestinal bleeding Patients with choledocholithiasis and cholangitis Patients with inflammatory bowel disease Patients with gastroenterological malignancy Patients with liver cirrhosis
Neurology	Patients with minor head trauma
Urology	Patients with a severe haematuria Patients with a terminal urologic disease
Otorhinolaryngology	Patients with a cuffed tracheal cannula Patients who need advanced airway care Patients with severe facial fractures
Geriatrics	Patients with severe delirium Patients who are terminal

### Intervention

#### Control group

Patients in the control group only received usual care. In usual care, physicians decided whether a patient could be discharged during the daily bedside rounds, using all the information usually available in the AAW, including medical history, medication use, and EWSs. The EWS was based on intermittently monitored vital signs: RR (based on a 30 s count by the nursing staff), HR, systolic blood pressure, oxygen saturation, oxygen supply, temperature, and level of consciousness. This monitoring was typically done every 8 h, or up to every hour if clinically deemed necessary.

#### Sensor group

In addition to receiving usual care, patients in the sensor group were equipped with an accelerometer-based wearable sensor (Healthdot, Philips Electronics BV, Eindhoven, The Netherlands). This validated sensor enabled continuous monitoring by measuring HR, RR, activity and posture every 5 min [11, 12]. During AAW stay, the data were accessible to the physicians and nurses via tables and figures within a dashboard (IntelliVue, Philips Electronics BV, Eindhoven, The Netherlands), which enabled them to conduct trend analyses for assessing patient stability. Furthermore, this dashboard also presented a score, which was similar to an EWS, but was solely based on the HR and RR measurements from the wearable sensor. This is described in more detail in the study protocol [10]. Physicians were offered to consider these continuous monitoring data, including their trends, during their daily bedside rounds when making discharge decisions, in addition to the information usually available. No predetermined protocol for consulting or interpreting the data was employed, allowing physicians to view and interpret the data according to their own judgment. After AAW discharge, wearable sensor data were no longer accessible for healthcare professionals, but were only collected, retrospectively. After 14 days, patients removed and discarded the wearable sensor. No alarms were used during the trial.

#### Outcomes

The primary outcome of the study was the difference in the proportion of the entire control and sensor groups that could be safely discharged home from the AAW. The concept of safe discharge was defined as the discharge of a patient from the AAW directly to home, thus without first being admitted to another in-hospital ward, and an absence of unplanned readmission, ED revisit, or mortality within the following 30 days. Secondary outcomes included LOS (AAW, in-hospital wards and overall hospital stay) and proportions of RRT calls and ICU admissions from the AAW. Data were collected from Electronic Medical Records (EMRs). Fourteen days after admission to the AAW, patients completed a questionnaire to assess readmissions to other hospitals.

### Statistical methods

Intention-to-treat analyses were used to evaluate the impact of continuous monitoring by the wearable sensor on the primary and secondary outcomes. Also, per-protocol analyses were conducted which excluded patients that failed to receive a functional sensor. The primary outcome was compared between the control and sensor group using a Chi-squared test. The secondary outcomes on LOS were compared using Mann–Whitney U tests due to non-parametric data. Differences in the proportions of RRT calls and ICU admissions from the AAW were compared using Fisher exact tests, as there were low incidences. Data analyses were done with Python (version 3.8). All tests used an α of 0.05 (two-sided).

The proportion of patients that is safely discharged from the AAW was hypothesized to increase from 40 to 50% by using continuous monitoring. Considering a power of 80%, an α of 0.05 (two-sided) and equal group sizes, a total of 768 patients were required to reject the null hypothesis. To account for a potential drop-out rate of 5%, 800 patients were intended to be included, with 400 patients in each group.

An interim analysis for futility was planned once 50% of the enrollment was completed. This interim analysis examined the likelihood that the sensor group would show the targeted ten percentage point improvement in safe discharge after enrollment was completed. For this purpose, the results of the already included sensor group patients were compared to hypothetical data of the to-be-included sensor group patients needed for this improvement (using a Chi-square test).

## Results

### Participants

From December 7th, 2021, until October 9th, 2023, 400 patients were randomized into a control and sensor group. A total of six patients (two in the control group and four in sensor group) withdrew from participation and were excluded from the analyses. Patients in the control (*n* = 198) and sensor (*n* = 196) group had comparable baseline characteristics (see Table 2). For instance, 51% were men in both groups, median age was 65 and 67 years and BMI 27.0 and 26.3 kg/m^2^, for the control and sensor groups, respectively.

**Table 2:** Baseline characteristics of patients in the control group and in the sensor group

	Control group (*n* = *198*)	Sensor group (*n* = *196*)
Age (years)	65 [53–74]	67 [52–74]
18–29	12 (6.1)	6 (3.1)
30–39	7 (3.5)	11 (5.6)
40–49	22 (11.1)	20 (10.2)
50–59	35 (17.7)	32 (16.3)
60–69	48 (24.2)	38 (19.4)
70–79	54 (27.3)	57 (29.1)
80 +	20 (10.1)	32 (16.3)
Male	101 (51.0)	100 (51.0)
BMI^a^ (kg/m^2^)	27.0 [24.1–30.7]	26.3 [23.4–30.0]
Manchester Triage System		
Non-urgent (blue)	0 (0.0)	1 (0.5)
Standard (green)	34 (17.2)	33 (16.8)
Urgent (yellow)	130 (65.7)	136 (69.4)
Very urgent (orange)	33 (16.7)	26 (13.3)
Immediate (red)	1 (0.5)	0 (0.0)
Specialism		
Pulmonology	62 (31.3)	63 (32.1)
Internal medicine	62 (31.3)	56 (28.6)
Surgery	39 (19.7)	28 (14.3)
Gastroenterology	22 (11.1)	25 (12.8)
Other	13 (6.6)	24 (12.2)
Time of AAW admission		
12.00–5.59 AM	23 (11.6)	27 (13.8)
6.00–11.59 AM	14 (7.1)	12 (6.1)
12.00–5.59 PM	78 (39.4)	68 (34.7)
6.00–11.59 PM	83 (41.9)	89 (45.4)
First MEWS in AAW	2 [0–4]	1 [0–4]
0–2	124 (62.6)	123 (62.8)
3–5	43 (21.7)	47 (24.0)
6–8	27 (13.6)	23 (11.7)
≥ 9	4 (2.0)	3 (1.5)
Diagnosis (ICD-10 code)^b^		
Pneumonia (J18)	20 (10.1)	18 (9.2)
COPD (J44)	13 (6.6)	11 (5.6)
Ileus (K56)	10 (5.1)	9 (4.6)
Other disorders of urinary system (N39)	8 (4.0)	8 (4.1)
Erysipelas (A46)	7 (3.5)	6 (3.1)
Kidney stones (N20)	4 (2.0)	8 (4.1)
Asthma (J45)	6 (3.0)	5 (2.6)
Abdominal and pelvic pain (R10)	4 (2.0)	7 (3.6)
Gastroenteritis (A09)	5 (2.5)	5 (2.6)
Lung cancer (C34)	4 (2.0)	6 (3.1)
Other	117 (59.1)	113 (57.7)

Values are given as median [interquartile range] or count (percentage). ^a^Was missing for 24 (12.1%) and 27 (13.8%) of the patients in the control and sensor groups, respectively. ^b^Top 10 most prevalent ICD-10 codes with basic description [13]. AAW: Acute Admission Ward, BMI: Body Mass Index, COPD: Chronic Obstructive Pulmonary Disease, ICD: International Classification of Diseases, MEWS: Modified Early Warning Score

### Primary outcome

**Fig. 1:**
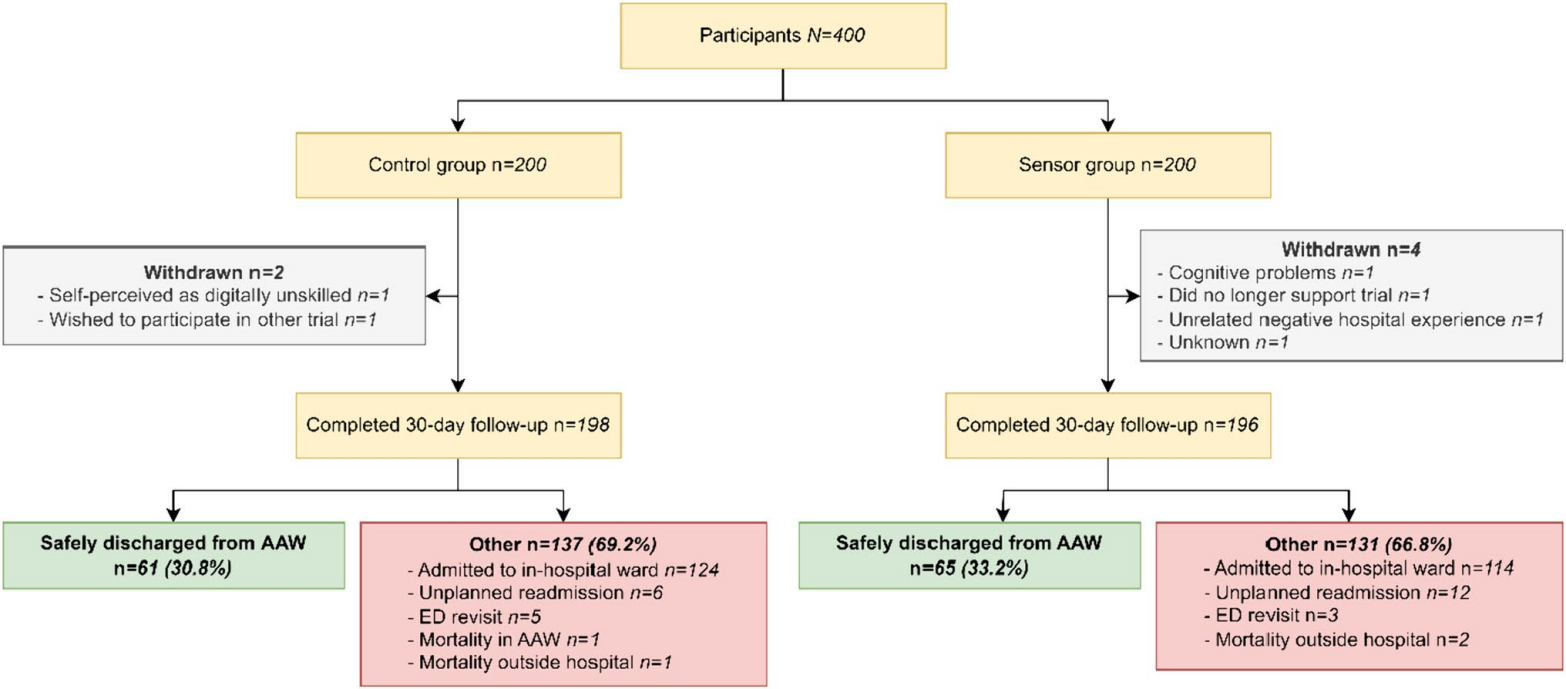
Participant flow and the proportions of patients that were safely discharged in the control and sensor group AAW: Acute Admission Ward, ED: Emergency Department

**Fig. 2:**
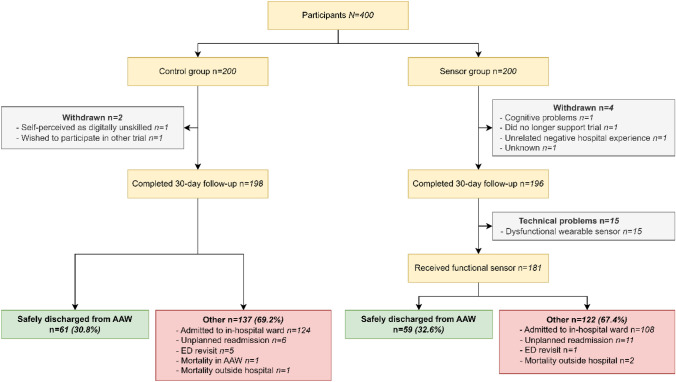
Participant flow and the proportions of patients that were safely discharged in the control and sensor group for the per-protocol analyses. Patients can only be in one category at a time, based on what happened first. However, if a patient visited the ED and was readmitted to the hospital within 30 days, this was only considered as a readmission. AAW: Acute Admission Ward, ED: Emergency Department

Figure 1 displays an overview of the primary outcome. The difference between the proportions of patients who were safely discharged from the AAW in the control (30.8%) and sensor (33.2%) group was not statistically significant (*p* = 0.62). Based on this outcome, it was indicated that the probability of detecting a significant difference would be exceedingly low if the trial were to be continued (*p* < 0.01, see Appendix 1). Due to the lack of effect, the trial was terminated prematurely. Furthermore, the difference between the proportions of patients who were admitted to an in-hospital ward after AAW stay in the control (124 out of 198 patients, 62.6%) and sensor group (114 out of 196 patients, 58.2%) was not statistically significant (*p* = 0.37). Per-protocol analysis showed similar results (see Appendix 1, Fig. 2).

Patients can only be in one category at a time, based on what happened first. However, if a patient visited the ED and was readmitted to the hospital within 30 days, this was only considered as a readmission.

### Secondary outcomes

Table 3 displays an overview of the secondary outcomes. Median LOSs for AAW stay, in-hospital ward stay and total hospital stay were lower in the sensor group compared to control group. However, these differences were not statistically significant nor deemed to be clinically relevant. An RRT call from the AAW occurred once. Similarly, one control group patient was admitted to the ICU from the AAW, while two sensor group patients were admitted to the ICU from the AAW. Per-protocol analysis showed similar results (See Appendix A, table 5).

**Table 3:** LOSs (hours), ICU admissions and RRT calls, in the control group and sensor group

	Control group *n* = *198*	Sensor group *n* = *196*	*p*-value
LOSs (hours)			
Hospital overall	83.5 [44.0–120.6]	68.4 [40.6–122.7]	0.31
AAW	24.4 [17.2–47.3]	22.7 [17.0–42.7]	0.52
In-hospital wards^a^	73.0 [46.9–142.2]	73.4 [46.3–142.6]	0.84
ICU admissions from AAW	1 (0.5)	2 (1.0)	0.62
RRT calls during AAW stay	1 (0.5)	0 (0.0)	1.00

Values are given as median [interquartile range] or count (percentage). ^a^Control group: *n* = 124,

sensor group: *n* = 114. LOS: Length Of Stay, ICU: Intensive Care Unit, RRT: Rapid Response Team

## Discussion

### Principal findings

This randomized controlled trial assessed the impact of adding continuous monitoring of HR and RR to usual care on the proportion of patients that can be discharged home safely directly from the AAW. These proportions did not differ significantly, as, respectively, 30.8% and 33.2% of the control and sensor groups were safely discharged home (*p* = 0.62). All secondary outcomes were also not statistically significantly different. However, the median LOS of total hospital stay was still higher in the control (83.5 h) than in the sensor group (68.4 h). This difference may be explained by the somewhat higher proportion of patients being admitted to an in-hospital ward in the control (62.6%) compared to the sensor group (58.2%) than by an effect of the wearable sensor. This is supported by having similar LOSs in the AAW (24.4 and 22.7 h, resp.) and in-hospital wards (73.0 and 73.4 h, resp.). The interim analyses indicated that the probability of detecting a significant difference in proportions of safely discharged patients would be exceedingly low if the trial were to be continued. Consequently, the trial was terminated prematurely.

### Interpretation of findings

The lack of effect of continuous monitoring on safe discharge may have multiple underlying causes. It may be that continuous monitoring does not exert any effect on safe discharge, because not the vital signs but other factors impede patient discharge. These factors may include: medical factors such as the requirement for intravenous medication which can only be given within the hospital, nursing factors such as the inability to provide homecare by caregivers, or logistical factors such as the inability to timely organize safe transport to home. Alternatively, it is possible that the continuously measured vital signs are valuable for detecting patient deterioration, but not patient stability. In other words, unstable vital signs are a reason for medical intervention or ICU admission, but stable vital signs are not the only reason for safe patient discharge. We did find, during a survey conducted among 32 physicians from the AAW (data not shown), that vital signs are considered highly important in assessing the patient, which is in line with literature [8]. Nevertheless, it is possible that in reality these values play a minor role in the discharge decision.

Still, it remains possible that the study was unable to detect an effect of continuous monitoring on safe discharge due to the design of the trial. The trial deliberately left the discharge decision to the attending physician, but failed to measure if and how the physician used the data for the discharge decision. It cannot be ruled out that some physicians did not look at the continuous data of HR and RR.

### Comparison with prior work

Literature on the impact of continuous monitoring using wearable sensors show variable findings. Some systematic reviews have concluded that in general there is currently insufficient statistical evidence to support its beneficial effects on clinical outcomes [6, 14]. However, it is noteworthy that certain studies have demonstrated a beneficial effect of continuous monitoring on LOS [15–17], as well as a reduction in the frequency of RRT calls and ICU admissions [15, 17–20]. Some even demonstrated a potential reduction in the likelihood of complications and mortality [21, 22]. Whether an effect is found or not, may depend on the specific device used (e.g. some devices measure additional vital signs) or the specific patient population.

For multiple reasons, it is challenging to determine whether positive effects of continuous monitoring on clinical outcomes exist. Firstly, most studies used a before-after design rather than a randomized controlled trial design, making these studies susceptible to confounding factors related to time. Secondly, different wearable devices were used across studies, which resulted in variations in the measured vital signs, extending beyond just HR and RR in certain cases, different measurement frequencies, and ways of data presentation to clinical staff. Thirdly, the studied populations were different, with a predominant focus on specific groups, particularly surgical patients. Since the value of continuous monitoring may differ between populations and use cases, this limits the generalizability to broad populations, such as those in AAWs.

### Strengths

To the best of our knowledge, this randomized controlled trial is the first to investigate the effects of continuous monitoring using wearable sensors on the discharge process of an AAW. This holds significant relevance considering the substantial number of patients admitted to AAWs on a daily basis. Furthermore, while the impact of continuous monitoring has been investigated in various specific surgical populations [10], this particular trial stands out as one of the few to explore patients from diverse medical specialties, thereby enhancing its potential generalizability to other patient populations. However, one could argue that this diversity diminishes the sensitivity in detecting discharge readiness, as the effect may vary significantly among different patient populations. Still, we believe that for all patients, even with differing diseases such as in AAW patients, stable vital signs remain important. Another noteworthy strength of this trial lies in the eligibility criteria, as patients were only included if their discharge destination following AAW admission was uncertain. This deliberate exclusion of patients with predetermined destinations ensures that the trial focuses on those cases where continuous monitoring could have a meaningful impact on the discharge process. Additionally, the randomized controlled design of the trial and the similarity in baseline characteristics between the control and sensor group allow for any observed differences in trial outcomes to be confidently attributed to the intervention.

### Limitations

Limitations of this trial may have contributed to the lack of effect observed. Firstly, the ED and the AAW both have a high workload and a substantial number of personnel with frequent turnovers, which posed challenges in training staff and keeping them updated. Hence, we have undertaken numerous training sessions and visited the ED and AAW multiple times per week, in an effort to ensure the staff was sufficiently updated and to enhance the inclusion process. However, because the inclusion had to be incorporated in the daily practice of the ED and AAW, the inclusion rate was slow and eligible subjects may have been missed. Also, because of this same high workload, it was considered infeasible to keep track of all eligible patients and their reasons for non-participation. Secondly, the slow inclusion rate resulted in a limited exposure of physicians to participants with a wearable sensor, which may have led physicians to forget to consider the continuous data in the discharge decision. Thirdly, physicians reported that logging in to a separate dashboard on the computer to access the measurements was time-consuming, potentially acting as a barrier to review the data during bedside rounds. A recent mixed methods trial [23] examined the implementation of continuous monitoring in two general wards and reported comparable challenges. It also emphasized the importance of seamless integration of the continuous measurements into the EMR to achieve effective intervention fidelity. Despite extensive efforts in our current trial, integration of the measurements within the EMR could not be achieved due to technical difficulties. Lastly, most physicians working at the AAW have limited experience in the interpretation of continuous data and trend analysis, which requires time to become accustomed to. Especially in the AAW, where clinical staff rotates frequently, this could pose a notable challenge, which may have further contributed to the lack of effect of continuous monitoring. Due to the lack of a reference standard on how to interpret the continuous data, physicians were not provided with a protocol on how to act upon the wearable sensor data.

Furthermore, there is a potential limitation in the trial’s design concerning the hypothesis to increase the proportion of safely discharged patients from 40 to 50%. Given that the discharge decision depends on a multitude of other factors besides HR and RR, this hypothesis may have been somewhat optimistic.

### Suggestions for future research

In light of the challenges encountered during the implementation of continuous monitoring in the course of this trial, we recommend that future studies direct their attention towards several key aspects. Firstly, careful consideration should be given to the accessibility of the collected data for nurses and physicians, preferably through seamless integration within the EMR framework. This may prevent an increased workload, which could otherwise impede the use of the acquired data. Following this, it would be of interest to investigate how continuous monitoring by wearable sensors could be implemented within hospital wards to potentially alleviate this workload. Second, efforts should be made to sufficiently expose nursing and medical staff to patients for whom continuous data on HR and RR is being collected. For example, by implementing continuous monitoring at an entire ward at once. This is important to sustain a heightened awareness regarding the availability of the collected data, thereby promoting its routine incorporation into clinical decision-making processes. Thirdly, future research might benefit from focusing on patients groups which are more dependent on the accurate assessment of patient stability or the prompt identification of signs of deterioration for informed clinical decision-making. Fourthly, it would be interesting to investigate whether continuous monitoring shortens the time elapsing between patient deterioration and its detection by healthcare professionals. Lastly, artificial intelligence may improve and automate the interpretation of continuous monitoring data for healthcare professionals. For instance, it could highlight specific patterns indicative of future patient deterioration. Additionally, artificial intelligence may combine continuous monitoring data with other hospital data to assess a patient’s discharge readiness.

## Conclusions

No significant effects of adding continuous monitoring to usual care on safe discharge, LOS, RRT calls, or ICU admissions in the AAW were found. The difficulties encountered in implementing continuous monitoring may have contributed to this lack of effect. Additional controlled trials are required to establish the clinical benefits of continuous monitoring in AAWs.

## Data Availability

The data analyzed in the current study are not publicly available due to legal and privacy considerations but are available from the corresponding author on reasonable request.

## Appendix: Additional analyses

### Interim analysis for futility

We assumed the proportion of safely discharged patients in the 200 to-be-included control group patients to be equal to that in the first 200 control group patients. This proportion is 30.8%. To obtain a 10 percentage points increase, which was deemed clinically relevant, the sensor group should obtain a proportion of safely discharged patients of 40.8%. To achieve this percentage, the to-be-included sensor group patients should be distributed as follows: 95 safely discharged and 101 not safely discharged patients. This was deemed unlikely to occur. Note that we assumed an equal dropout rate and no technical problems as most technical problems happened in the first months of the trial.

To compare the to-be-included sensor group patients with the currently obtained sensor group patients, a Chi-square test resulted in *p* < 0.01. The corresponding contingency table is shown in Table

**Table 4:** Contingency table used for the interim analysis for futility

	Safe discharge	Other	Total
Already included sensor group patients	65	131	196
To-be-included sensor group patients	95	101	196
Total	160 (40.8%)	232 (59.2%)	392 (100%)

4.

### Per-protocol analyses

In Fig. 2, the main outcome is illustrated for the per-protocol analyses. The proportion of patients in the sensor group that was safely discharged (32.6%) and was admitted to an in-hospital ward (59.7%) were similar to the proportions obtained in the intention-to-treat analyses (33.2% and 58.2%, resp.). The proportion of safely discharged patients in the control group did not change compared to the intention-to-treat analyses (30.8%), as all patients in the control group received the care as aimed for in the control group (See Table

**Table 5:** LOSs (hours), ICU admissions and RRT calls, in the control group and sensor group

	Control group *n* = *198*	Sensor group *n* = *181*	*p*-value
LOSs (hours)			
Hospital overall	83.5 [44.0–120.6]	69.0 [42.1–123.1]	0.48
AAW	24.4 [17.2–47.3]	22.8 [17.0–43.6]	0.55
In-hospital wards^a^	73.0 [46.9–142.2]	72.9 [46.1–142.1]	0.90
ICU admissions	1 (0.5)	2 (1.0)	0.61
RRT calls	1 (0.5)	0 (0.0)	1.00

Values are given as median [interquartile range] or count (percentage).

^a^Control group: *n* = 124, sensor group: *n* = 108. ICU: Intensive Care Unit, LOS: Length Of Stay, RRT: Rapid Response Team

5).
